# Urban Stream Degradation, Organic Matter Retention and Implications for Environmental Health in the Central Amazon

**DOI:** 10.3390/ijerph23040418

**Published:** 2026-03-26

**Authors:** Sthefanie Gomes Paes, Joana D’Arc de Paula, Luis Paulino da Silva, Vanessa Campagnoli Ursolino, Maria Teresa Fernandez Piedade, Aline Lopes

**Affiliations:** 1 Programa de Pós-Graduação em Ecologia, Instituto Nacional de Pesquisas da Amazônia, Manaus 69067-375, AM, Brazil; sthefaniegsouza@gmail.com; 2 Grupo de Ecologia, Monitoramento e Conservação de Áreas Úmidas, Instituto Nacional de Pesquisas da Amazônia, Manaus 69067-375, AM, Brazil; jddpaula@gmail.com (J.D.d.P.); maitepp@inpa.gov.br (M.T.F.P.); 3 Laboratório de Ecologia, Universidade Nilton Lins, Manaus 69058-030, AM, Brazil; biologicas63@gmail.com; 4 Programa de Pós-Graduação em Tecnologias Limpas (PPGTL), Universidade Cesumar, Maringa 87050-900, PR, Brazil; campagnolivanessa1@gmail.com; 5 Instituto Cesumar de Ciência Tecnologia e Inovação (ICETI), Universidade Cesumar, Maringa 87050-900, PR, Brazil

**Keywords:** public health, nature-based solutions, riparian forests, urban water pollution, water resources

## Abstract

**Highlights:**

**Public health relevance—How does this work relate to a public health issue?**

- Urban streams with low organic matter retention are more vulnerable to pollution accumulation, directly affecting water quality and increasing health risks for surrounding populations.
- The degradation of riparian vegetation in urban catchments contributes to sedimentation, waste accumulation, and reduced ecosystem services, which are closely linked to urban environmental health problems.

**Public health significance—Why is this work of significance to public health?**

- The study demonstrates how urbanization alters hydrological and ecological processes that regulate water quality, with direct implications for disease transmission, sanitation, and environmental degradation.
- By documenting low leaf retention capacity and high levels of solid waste and pollution, the research highlights structural weaknesses in urban watershed management that affect population well-being.

**Public health implications—What are the key implications or messages for practitioners, policy makers and/or researchers in public health?**

- Urban stream restoration and riparian buffer recovery should be incorporated into public health and urban planning policies as preventive strategies to improve environmental quality and reduce health risks.
- Integrated watershed management is essential for mitigating pollution, controlling runoff, and enhancing ecosystem services that support healthy urban environments.

**Abstract:**

Urbanization alters the hydrological and structural functioning of tropical urban streams, influencing organic matter transport and retention processes. This study investigated leaf litter retention dynamics in the Bindá Stream in central Amazonia. A six-month leaf release experiment (100 leaves per 12 trial; 1200 leaves total) was conducted alongside hydrological monitoring and floristic surveys of riparian vegetation (adult and regeneration strata). Leaf retention remained consistently low (<33%) across sampling periods. Generalized linear models indicated that flow velocity and discharge were the primary predictors of retention probability, with higher hydrodynamic intensity significantly reducing in-stream storage. Riparian vegetation exhibited moderate structural complexity (Shannon H′ = 1.80; Structural Complexity Index = 3.80), yet limited channel roughness and physical obstructions constrained retention efficiency. Anthropogenic debris locally increased retention, but represents a structurally altered retention mechanism. Hydrodynamic forcing, rather than precipitation totals alone, governed organic matter transport dynamics. Reduced retention capacity suggests limited buffering of downstream material export under high-flow conditions. Although direct water-quality or epidemiological indicators were not measured, findings align with ecohydrological frameworks linking structural simplification and flow flashiness to diminished ecosystem regulation. These results inform riparian restoration and urban stormwater management strategies aimed at enhancing ecosystem regulation and water-quality buffering in tropical cities.

## 1. Introduction

Urban streams are increasingly recognized as critical socio-ecological systems linking watershed integrity, ecosystem functioning, and public health outcomes [1]. In tropical cities, where rapid and often unplanned urbanization overlaps with high hydrological seasonality, degradation of headwater streams may influence environmental conditions associated with pathogen exposure, flood risk, and water security [1,2,3]. While the Amazon Basin is globally known for its hydrological importance, recycling between 24% and 35% of its water annually and contributing approximately 6400 km^3^ year^−1^ to atmospheric moisture through “aerial rivers” [4], far less attention has been given to how urban degradation of small headwater streams (*igarapés*) affects ecosystem functioning and environmental health at the city scale [1].

Headwater streams represent up to 90% of the Amazon basin’s drainage network [5] and play a disproportionate role in regulating nutrient transport, organic matter processing, and downstream water distribution. In forested conditions, these streams are oligotrophic, acidic (pH 4.0–5.0), and ion-poor (<20 μS cm^−1^), reflecting the geological characteristics of the Alter do Chão formation underlying Manaus [6,7]. Nutrient retention occurs primarily within the organic compartment of the forest, rather than in mineral soils, creating tight biogeochemical cycling and minimal downstream export [8].

Urbanization disrupts this equilibrium through channelization, riparian deforestation, impervious surface expansion, and the discharge of untreated sewage, processes collectively described as the Urban Stream Syndrome and, in the Global South, the Southern Urban Hydrosystem Syndrome (SUHS) [1,9]. Unlike temperate systems, where stormwater runoff dominates impacts, SUHS is strongly associated with continuous sewage discharge and solid waste accumulation [1,9]. In Manaus, approximately 40% of residents discharge effluents directly into streams [7], and up to 90% of wastewater in Amazonian urban centers remains untreated [10].

These alterations fundamentally modify hydrology and organic matter dynamics. Impervious cover generates “flashy” hydrographs with rapid discharge peaks and reduced baseflow stability [9]. Removal of riparian vegetation eliminates structural elements such as large woody debris and root mat complexes that govern organic matter retention and nutrient dynamics. Experimental removal of riparian woody vegetation has been shown to substantially alter stream chemistry and production–respiration dynamics [11], and conceptual frameworks highlight reductions in organic matter retention and geomorphological simplification following riparian loss. Retention of leaf litter is a key functional process in headwater streams because allochthonous inputs constitute the primary energy source for aquatic food webs [12].

Experimental studies in Central Amazonia demonstrate that litter breakdown rates are significantly faster in minimally urbanized streams, where fungal biomass and shredder abundance remain high [13]. In contrast, urban streams exhibit a threefold reduction in litter breakdown and severe disruption of macroinvertebrate-mediated energy transfer [14]. However, while breakdown processes have been studied, the specific role of physical retention capacity under urban hydrological alteration remains underexplored, particularly in relation to public health implications.

Urban-induced loss of retention capacity may have cascading consequences. Reduced retention accelerates downstream transport of organic material and nutrients, potentially contributing to eutrophication, hypoxia, and microbial proliferation in receiving waters [14,15]. Additionally, accumulation of leaves and anthropogenic debris in channelized reaches may create stagnant microhabitats favorable to vector mosquitoes and waterborne pathogens [1]. Despite these documented ecological mechanism links, few studies in tropical urban systems have explicitly connected alterations in organic matter retention to environmental health risks.

The Central Amazon streams provide a critical case study for examining these linkages. Urban streams in Manaus show drastic biodiversity loss, including a 91% decline in macroinvertebrate richness in urban reaches compared to preserved forest streams [3], homogenization of fish assemblages [1,16], and elevated heavy metal concentrations exceeding environmental standards in industrial tributaries such as Igarapé do Quarenta [7]. Bioaccumulation studies further demonstrate human exposure risks via fish consumption and dermal contact [7].

Yet, while chemical contamination and biodiversity loss have been documented, the functional dimension of organic matter retention, and its integration with hydrological alteration and environmental health, remains insufficiently quantified in Amazonian urban headwaters [7,17]. Thus, this study addresses that gap by investigating leaf litter transport and retention dynamics in the Bindá Stream, an urbanized headwater within the São Raimundo watershed in Manaus. We integrate hydrological monitoring, obstacle frequency assessment, and riparian vegetation analysis to evaluate how urbanization modifies retention capacity.

We hypothesize that (1) reduced riparian complexity and increased flow variability are expected to constrain organic matter retention; (2) anthropogenic debris partially compensates for natural retention but creates artificial retention structures; and (3) diminished retention may reduce the buffering capacity of urban streams, potentially enhancing downstream material export under high-flow conditions.

By linking ecosystem functioning to hydrological alteration and public health relevance, this study contributes to the growing international literature on tropical urban streams and provides an evidence-based framework applicable to rapidly urbanizing regions in Southeast Asia, Sub-Saharan Africa, and Latin America.

## 2. Materials and Methods

### 2.1. Study Area

**Figure 1:**
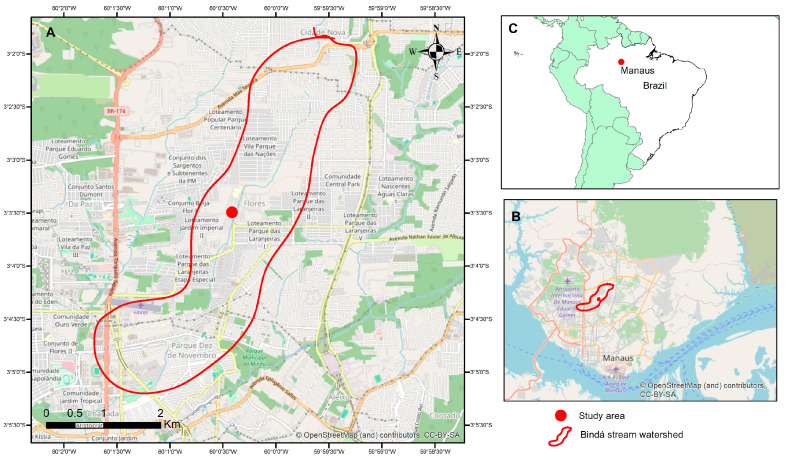
Location of the Bindá stream watershed in Manaus, Amazonas, Brazil. (**A**) Detailed map of the Bindá stream watershed (red line) showing the surrounding urban landscape in the central-northern region of Manaus. The red dot indicates the specific study area within the Nilton Lins University campus. (**B**) Map of the city of Manaus highlighting the location of the Bindá stream watershed. (**C**) Geographic location of Manaus in northern Brazil.

The study was conducted in a Permanent Preservation Area (PPA) located in the middle section of the Bindá Stream watershed. This area lies within the campus of Nilton Lins University in Manaus, Amazonas State, Brazil (Figure 1). The Bindá Stream originates in the Mundo Novo neighborhood (northern zone) and flows toward the Chapada neighborhood (central-southern zone), forming a second-order watershed. The watershed covers approximately 10 km^2^ and has a perimeter of 20 km [3]. The main channel of the stream is 8.4 km long, with a total drainage network of 14.0 km, exhibiting a predominantly dendritic pattern [18]. The catchment is highly urbanized, with impervious surfaces, channel modifications, and fragmented riparian vegetation, which influence hydrological responses and organic matter dynamics [19,20].

Due to urbanization, the stream’s riparian vegetation has been extensively altered by land use and occupation. The riparian zone is narrow, ranging from 5 to 10 m in width. Some sections have been modified with stormwater drainage pipes and concrete banks aimed at reducing sedimentation. Upstream of the study reach, the channel is impacted by sewage discharge and solid waste accumulation, resulting in changes to turbidity, acidity, conductivity, and dissolved oxygen [3].

The local vegetation consists of regenerating secondary forest [3], with about 75% of the tree species being fruit-bearing and non-native, planted after initial deforestation. Dominant families include Fabaceae, Anacardiaceae, and Myrtaceae. The stream banks are also lined with *Roystonea oleracea* (Arecaceae), an exotic palm species.

### 2.2. Vegetation Sampling and Data Collection

Riparian vegetation was surveyed along the 30 m study reach to assess floristic composition and structural attributes. All tree and shrub species were identified, and their origin (native, introduced, or naturalized) and vertical stratum (adult and/or regeneration) were recorded. In addition, herbaceous species present along the stream margins were documented to characterize seasonal changes in lateral vegetation cover during the study period.

Vegetation data were collected through systematic walks along the banks of the *igarapé* (stream), following established protocol [21]. This method enabled the identification and recording of the riparian vegetation types present in the study area. Species richness was assessed by compiling a list of all recorded plant species, which were classified according to vegetation strata, based on consultations with the Reflora Virtual Herbarium database.

This floristic survey was conducted solely to characterize the structural and compositional context of the study reach where the leaf retention experiments were performed, and not to establish direct statistical relationships between vegetation attributes and retention rates. Vegetation was documented to provide ecological context, as riparian structure influences organic matter inputs and ecosystem services related to water quality and habitat provision [13,20].

### 2.3. Experimental Design

**Figure 2:**
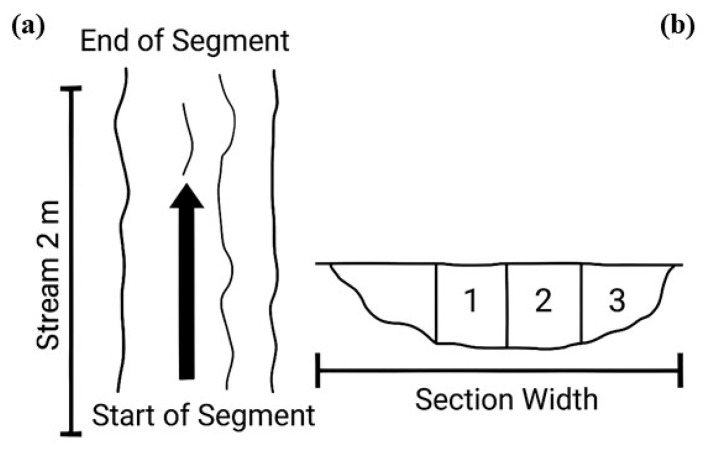
Illustration of the stream section analyzed. (**a**) schematic representation of the 2 m segments used in the leaf transport experiment. The arrow indicates the direction of water flow; (**b**) diagram of depth measurements within each segment for stream discharge estimation.

The experiment took place during the rainy season, from November 2017 to April 2018, with two sampling events per month, totaling 12 observations. We used the leaf release and capture method to assess leaf litter transport and retention in a 30 m open stream segment, subdivided into 15 sections of 2 m each (Figure 2a), following established protocols [22].

For each segment, physical habitat variables were recorded, including water depth, channel width, flow velocity, and discharge. Site characterization also involved noting the presence of obstacles such as marginal vegetation, fallen logs, sandbanks, and solid waste. Although the segment boundaries remained fixed, recharacterization was necessary for each sampling due to natural potential changes.

The depth and width of the stream segment were determined using a polyvinyl chloride (PVC) pipe marked with intervals, functioning similarly to a measuring tape. During each observation, the flow velocity of each study segment was measured (Figure 2b). Flow velocity refers to the speed of the current per unit area and is expressed in meters per second (m s^−1^). Typically, instruments such as current meters or flowmeters, small propellers that rotate as water passes through, are used to measure velocity. However, in contexts with shallow water depth and limited access to equipment or resources, as in the present study, indirect measurement techniques offer a reliable alternative [23], such as the float method [24].

Discharge (Q, in m^3^ s^−1^) was calculated as the product of the average velocity (corrected by a factor of 0.8) and the average cross-sectional area. The stream cross-section was divided into vertical intervals, and the depth of each was measured. The total area (TA) was computed as the sum of individual segment areas:

$${TA}_{\equiv \sum_{i=1}^{n}A_{i}}$$

where TA is the area of vertical segment i, and n is the total number of intervals.

During each experimental trial, basic physicochemical parameters were measured in situ to characterize ambient stream conditions. Water temperature (°C), electrical conductivity (µS cm^−1^), dissolved oxygen (mg L^−1^), and pH were recorded using a portable multiparameter probe (previously calibrated according to manufacturer specifications). Measurements were taken at mid-channel, approximately 10 cm below the water surface, immediately prior to leaf release, to ensure that values reflected baseline conditions during each transport trial. These measurements were used to describe the physicochemical context under which leaf retention dynamics were evaluated, but were not incorporated as explanatory variables in the statistical models.

### 2.4. Obstacle Classification and Rainfall Data

Anthropogenic and natural obstacles were categorized into low, intermediate, and high frequency based on the number of obstacles per 10 m reach. Obstacles were visually classified based on their interference with leaf transport. Obstructions affecting ≤30% of the channel were categorized as low frequency, 30–70% as intermediate, and >70% as high frequency. Water column height above each obstacle was measured using the PVC rod.

Rainfall data for the study period were obtained from the Brazilian National Institute of Meteorology [25].

### 2.5. Leaf Retention Experiment

A total of 100 naturally fallen leaves were used per trial, across 12 experimental trials conducted between November 2017 and April 2018, totaling 1200 leaves. Leaves were collected immediately prior to each trial from both stream banks within the study reach to ensure they represented locally available allochthonous input.

The tracer material consisted primarily of dominant broad-leaved coriaceous riparian species, composed of approximately 70% *Cecropia manaura* Aguiar, Demarchi & Piedade and 30% *Vismia japurensis* Reichardt. These species were selected because they represented the most abundant naturally occurring leaf litter types within the study reach, thereby increasing ecological realism and reproducibility.

Leaves were lightly marked with non-toxic, waterproof, biodegradable paint to enable visual tracking during transport. To reduce excessive buoyancy and simulate natural litter saturation, leaves were submerged in stream water for 12 h prior to release [26]. This preconditioning step is commonly employed in tropical stream retention studies to approximate natural leaf hydration status. The marking procedure was applied superficially and did not alter leaf flexibility or morphology; any potential influence on transport dynamics is considered negligible relative to hydrodynamic forcing.

**Figure 3:**
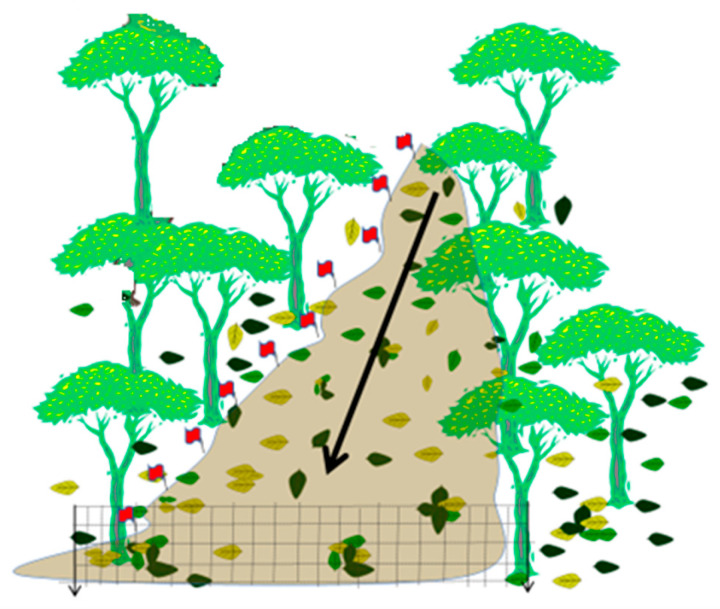
Schematic representation of the leaf release and capture experiment conducted along the 30 m study reach. Leaves were released at standardized upstream points, and their downstream transport or in-stream retention was monitored at the terminal sampling station. After a two-hour interval, all retained leaves were counted and collected within the 15 predefined channel segments. The red flags indicate the specific locations where the measurements were taken. The arrow indicates the direction of water flow. Retention percentage was calculated for each trial as the proportion of released leaves remaining within the reach, and results are expressed as mean ± standard deviation.

For each trial, leaves were released simultaneously at a standardized upstream point immediately above the 30 m study reach. The reach was subdivided into 15 consecutive 2 m segments. A mesh net was installed at the downstream end to intercept transported (non-retained) material (Figure 3).

After a two-hour interval, all retained leaves within each segment were counted and collected. Leaves intercepted by the downstream net were classified as transported. Retention percentage was calculated for each trial as: Retention (%) = (Number of retained leaves/100) × 100.

Although experimental leaf marking may slightly modify surface properties, this approach is widely adopted in fluvial ecological research to evaluate retention dynamics under controlled and replicable conditions [26,27,28,29,30,31]. The method is considered appropriate for assessing relative retention efficiency within small tropical urban streams.

### 2.6. Statistical Analysis

Leaf transport probability along the 30 m reach was analyzed using survival analysis techniques. Differences between transport curves were tested using the non-parametric log-rank (LR) test, which accommodates non-linear increases in retention probability over distance [27,28]. In the survival framework, retained leaves were treated as events, whereas transported leaves were considered right-censored observations. The log-rank statistic was calculated as:

$$LR=\;\frac{{\left(d_{1}{-\;E}_{1}\right)}^{2}}{E_{1}}+\frac{{\left(d_{2}-E_{2}\right)}^{2}}{E_{2}}.$$

where *d* is the observed number of retained leaves at interval i, and E1 is the expected number of deaths in population 1 during interval i; E2 is the expected number of deaths in population 2 during interval i.

The log-rank test was used to compare transport curves based on obstacle frequency, free water column height, and discharge.

To evaluate monthly variation in physical and hydrological variables (width, depth, flow velocity, discharge, and rainfall), one-way ANOVA (95% confidence level) was performed after verifying assumptions of normality (Shapiro–Wilk test) and homoscedasticity. When necessary, data were log-transformed to meet model assumptions. The independent variable was sampling month, and the dependent variables were stream characteristics.

To further quantify the influence of hydrological drivers on retention probability, generalized linear models (GLMs) with binomial error distribution and logit link function were applied. The response variable was specified as the number of retained versus transported leaves per trial (cbind (Retained, Transported)). Separate models were fitted, including flow velocity and discharge as predictors. These models allowed direct estimation of the effect of increasing hydraulic forcing on leaf retention probability.

Additionally, Spearman’s rank correlation analyses were conducted to assess monotonic relationships between retention rate and velocity, discharge, and precipitation. This non-parametric approach was selected due to the small sample size and the presence of tied values.

Riparian vegetation structural complexity was quantified using standard ecological diversity metrics calculated separately for the adult tree stratum and the regeneration (shrub) stratum. Species abundance data obtained during the floristic inventory were used to compute Shannon diversity (H′), Simpson diversity (1–D), and Pielou’s evenness (J). Shannon diversity (H′) was calculated to estimate floristic heterogeneity, Simpson diversity (1–D) to evaluate dominance patterns, and Pielou’s index (J) to assess the distribution uniformity of individuals among species within each stratum.

To characterize overall riparian structural complexity within the study reach, a Structural Complexity Index (SCI) was derived by combining total Shannon diversity (considering both strata) with the number of vertical strata present in the vegetation profile. This composite index was used as a proxy for vertical and compositional heterogeneity in the riparian corridor.

Because vegetation structure was surveyed once and remained constant during the experimental period, these indices were used to describe the structural context of the study reach rather than as temporal predictors in inferential statistical models of leaf retention.

All statistical analyses were performed in R 4.3.3 (R Core Team, 2023), using the survival package for log-rank tests, the vegan package for diversity indices, and base R functions for GLMs and correlation analyses.

### 2.7. Limitations

We acknowledge some limitations associated with the study design.

First, although the marking procedure was superficial and non-toxic, it may have slightly altered leaf surface properties or buoyancy, potentially influencing transport dynamics. However, this method is widely used in retention experiments and was applied consistently across trials [29,30,31,32].

Second, flow velocity estimates were derived from surface float measurements, which provide an approximation of hydrodynamic conditions and may not fully capture vertical or lateral flow heterogeneity within the channel.

Third, although the experiment included 12 trials totaling 1200 leaves, all assays were conducted during the rainy season. During the dry season, discharge was insufficient to permit leaf transport. Consequently, the results represent retention dynamics under high-flow conditions and do not capture seasonal contrasts or interannual variability.

Finally, while replication was adequate to detect spatial retention patterns within the study period, it does not encompass the full range of intra- and inter-storm hydrological variability.

These constraints should be considered when interpreting the ecological and public health implications of leaf retention and organic matter dynamics in this urban tropical stream.

## 3. Results

### 3.1. Hydrological Dynamics and Seasonal Patterns

During the experimental period, physicochemical parameters reflected degraded urban conditions, with elevated conductivity (407 µS cm^−1^), low dissolved oxygen (0.73 mg L^−1^), a mean temperature of 30.9 °C, and a pH of 5.9.

Mean discharge and flow velocity varied significantly among months (ANOVA, *p* < 0.05), with the highest velocities recorded during peak rainfall months (November–December; Table 1).

Although December recorded the highest rainfall, stream depth and discharge were comparatively lower during baseflow measurements conducted 48–72 h after rainfall events. This rapid response is typical in urbanized catchments with high impervious surfaces, promoting flash floods followed by quick recession. Overall, instantaneous hydraulic conditions (velocity and discharge) varied independently from total precipitation magnitude during the experimental window.

### 3.2. Riparian Vegetation Composition and Seasonal Variation

The riparian vegetation along the Bindá Stream included species from three distinct strata: arboreal, shrub (regeneration), and herbaceous. In the arboreal stratum, eight species were recorded, distributed among eight genera and four families. Of these, seven were native to Brazil and one was naturalized (*Psidium guajava* L.). The most representative families were Fabaceae (three species), Anacardiaceae (two species), and Myrtaceae (two species).

The shrub stratum also comprised eight species across eight genera and five families. Some species were found in both arboreal and regeneration strata (e.g., *Inga edulis*, *Psidium guajava*, *Syzygium cumini*, and *Euterpe oleracea*), while others were exclusive to the regeneration layer, such as *Mauritia flexuosa*, *Syzygium malaccense*, and *Genipa americana*. Conversely, species like *Clitoria fairchildiana* and *Senna alata* were observed only in the adult tree stratum. Detailed taxonomic and ecological information is presented in Table 2.

In the herbaceous stratum, only one species (*Pennisetum purpureum* Schumach., Poaceae) was recorded during the dry season. In contrast, the rainy season yielded 13 herbaceous species, reflecting a notable increase in richness. These were mainly from the families Poaceae, Cyperaceae, and Euphorbiaceae. Three species (*Commelina erecta* L., *Cyperus odoratus* L., and *Mimosa pudica* L.) were recorded in both seasons. In certain stretches of the stream, channel sections were found to be impervious and lacked adjacent riparian vegetation, potentially affecting the structure and composition of local vegetation. The seasonal distribution of herbaceous species is summarized in Table 3.

The adult tree stratum exhibited moderate floristic diversity (Shannon H′ = 1.50), with a Pielou evenness index of 0.72 and Simpson diversity (1–D) of 0.71. These values indicate a community with moderate heterogeneity but noticeable structural dominance.

The regeneration stratum showed slightly higher diversity (H′ = 1.66), with greater evenness (J = 0.80) and Simpson diversity (1–D = 0.79), contributing to increased vertical heterogeneity.

When both strata were combined, total Shannon diversity reached H′ = 1.80. The Structural Complexity Index (SCI = 3.80) reflects a riparian system with moderate structural complexity.

Although species origin (native, introduced, naturalized) was recorded, structural contribution rather than biogeographic status was considered functionally relevant for retention dynamics. Introduced species such as *Syzygium cumini* and *Mangifera indica* contributed to basal area and regeneration density, thereby influencing physical roughness independent of origin.

Vegetation data were used descriptively to characterize the physical template within which hydrodynamic processes operated. Because vegetation structure remained temporally constant during the six-month experimental period, it was not modeled as a time-varying predictor but interpreted as a background structural condition influencing flow–retention interactions.

### 3.3. Leaf Retention Dynamics

**Figure 4:**
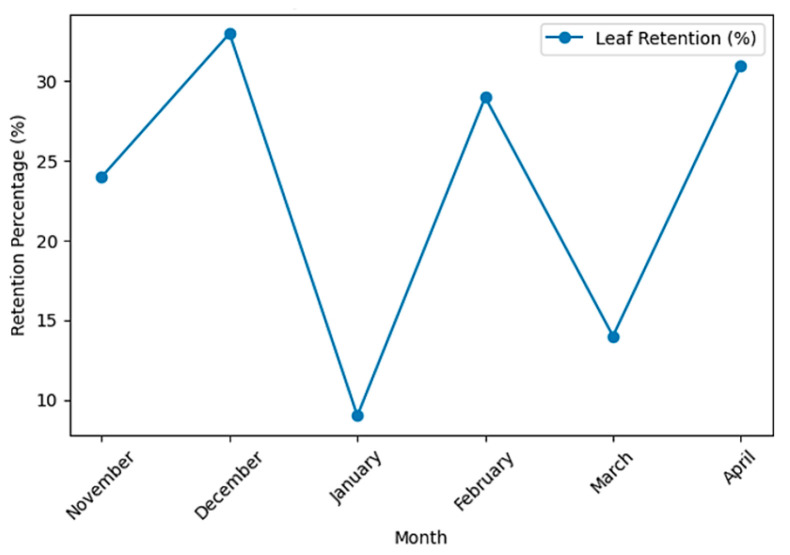
Monthly variation in the proportion of leaves retained along a 30 m reach of the Bindá Stream between November 2017 and April 2018. Each experimental event consisted of the release of 100 leaves under rainy-season flow conditions.

Leaf retention remained low throughout the experimental period, ranging from 9% (January) to 33% (December). Of the 1200 leaves released across 12 trials, 277 were retained within the 30 m reach, corresponding to an overall retention rate of 23.1% (Figure 4).

**Figure 5:**
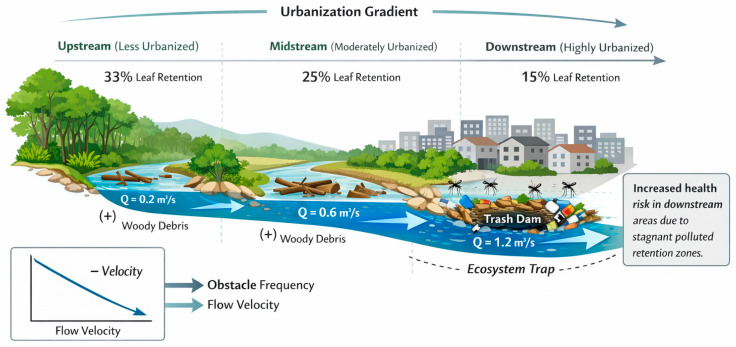
Conceptual spatial model illustrating hydrological gradients, obstacle frequency, and leaf retention patterns along the urban stream continuum in Bindá stream, Manaus, Brazilian Amazon. The arrows indicates the direction of water flow.

Leaf transport exhibited a consistent decline in retention probability with increasing distance from the release point. Across most trials, more than 65% of released leaves were transported beyond the study reach. Transport distance was strongly influenced by channel structure. In segments with low obstacle frequency, leaves frequently traveled 25–30 m, whereas in high-obstacle segments, transport distances were reduced to 10–15 m (Figure 5).

**Figure 6:**
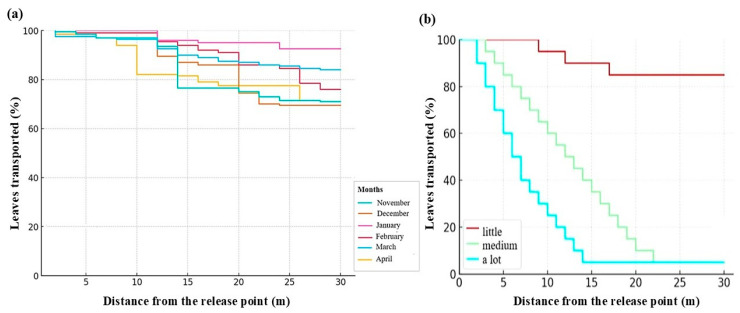
(**a**) Proportion of leaves transported across a 30 m reach of the Bindá Stream between November 2017 and April 2018 (200 leaves released per month); (**b**) Leaf transport as a function of obstacle frequency.

Log-rank tests revealed significant differences in transport curves among months (χ^2^ = 107, *p* < 0.001), obstacle frequency (χ^2^ = 16.8, *p* = 0.0002), unobstructed water column height (*p* < 0.001), and discharge (*p* < 0.001). January and March exhibited the highest transport rates, whereas December and April showed comparatively greater retention (Table 4; Figure 6).

At the monthly scale (n = 6), multiple linear regression including discharge and precipitation was statistically significant (F(2,3) = 11.65, *p* = 0.039). Discharge exerted a significant negative effect on retention (β = –54.35, *p* = 0.020), while precipitation was not significant after accounting for hydraulic response. Due to limited temporal replication, these results should be interpreted as exploratory.

Generalized linear models (binomial error distribution) performed at the leaf level demonstrated significant negative effects of velocity (β = –4.21 ± 1.04 SE, *p* < 0.001) and discharge (β = –5.05 ± 0.84 SE, *p* < 0.001) on retention probability.

Spearman’s rank correlations indicated negative but non-significant associations between retention rate and velocity (ρ = –0.41, *p* = 0.42) and discharge (ρ = –0.77, *p* = 0.10), reflecting limited monthly replication (n = 6).

Together, these analyses indicate that hydraulic forcing, particularly velocity and discharge, was the primary driver of leaf transport within the study reach.

### 3.4. Obstacle Frequency and Anthropogenic Debris

**Figure 7:**
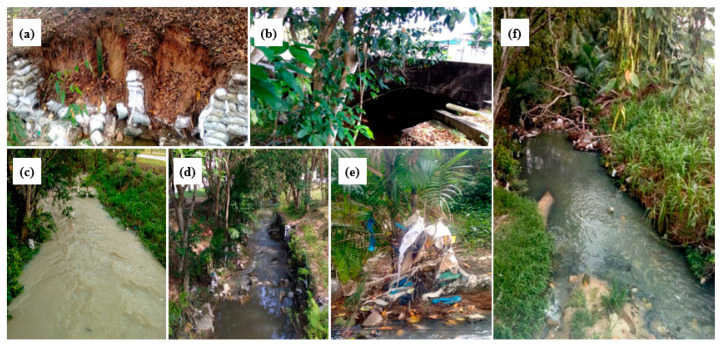
Bindá Stream between November 2017 and April 2018, Manaus, Amazonas, Brasil. (**a**) soil erosion along the banks; (**b**) stormwater discharge channel; (**c**) high water volume on a rainy day; (**d**) reduced water volume on a dry day; (**e**) solid waste and riparian vegetation acting as obstacles to leaf retention; (**f**) signs of water pollution, siltation, and solid waste accumulation.

Obstacles were categorized consistently as low, intermediate, or high frequency, correcting previous inconsistencies. Both natural elements (e.g., woody debris) and anthropogenic materials (e.g., plastic waste) contributed to localized leaf retention (Figure 7).

Artificial debris partially compensated for the reduced availability of natural retention structures but does not replicate the ecological functions of coarse woody material. Field observations documented bank erosion, stormwater discharge inputs, and seasonal fluctuations in water volume (Figure 7a–f).

These structural conditions influenced local retention patterns but did not override the dominant effect of hydraulic forcing.

## 4. Discussion

Riparian vegetation along the 30 m study reach exhibited moderate structural complexity (SCI = 3.80), reflecting a common pattern in lotic ecosystems under urban influence, where moderate structural complexity is often accompanied by reduced alpha diversity compared to pristine areas, as observed in riparian fragments of the Atlantic Forest in Southeastern Brazil [33]. The observed Shannon diversity (H′ = 1.80) situates the stream in an intermediate conservation stage, typical of urban fragments subjected to selective pressures.

Partial dominance in the adult canopy (H′ = 1.50; J = 0.72), driven by a few abundant taxa, suggests a “biological narrowing” effect. In this process, a limited number of pioneer or stress-tolerant species establish and dominate the upper stratum, a pattern consistent with observations in fragmented tropical savannas (Cerrado) [34]. In contrast, the higher evenness (J = 0.80) and diversity (H′ = 1.66) in the regeneration layer indicate active successional dynamics. Vertical heterogeneity and increased lateral vegetation density are functionally critical; dense marginal strata act as physical barriers that increase hydraulic roughness and enhance organic matter retention, even in disturbed agricultural or urban catchments [35].

In urban contexts, characterized by “flashy” flows, the lateral density of regeneration layers may stabilize streambanks and mitigate excessive debris transport during storm events. Nevertheless, environmental quality remains severely compromised: dissolved oxygen below 1 mg L^−1^ and conductivity exceeding 400 µS cm^−1^ indicate intense organic enrichment, likely linked to sewage inputs [7]. These conditions suggest that the stream functions more as a degraded conduit than a healthy biological filter.

Structural complexity influences leaf retention by increasing hydraulic roughness and promoting flow deceleration, facilitating the formation of small-scale retention structures such as root mats and low-hanging branches [36,37,38]. Our findings support the hypothesis that riparian fragments with simplified vegetation retain less organic matter, a trend consistent with the ‘urban stream syndrome’ [39,40]. This syndrome is characterized by reduced connectivity between the riparian zone and the channel, which diminishes coarse particulate organic matter (CPOM) storage and impairs nutrient processing [41]. Furthermore, the increasing presence of exotic and cultivated species likely alters the quality and phenological timing of organic inputs, further disrupting the natural energy flow of the system [42].

Regression analysis indicated that water flow was the main driver of leaf retention, while precipitation had a weaker influence. Even during peak rainfall months (e.g., December), low stream depth and discharge values were recorded, suggesting that rainfall primarily contributes to rapid surface runoff (flashy flows) with minimal groundwater recharge [43,44]. Thus, hydraulic energy, rather than cumulative rainfall volume, determines retention outcomes in urbanized tropical streams. Between January and March, higher transport was linked to increased discharge and a lack of physical obstructions. Conversely, retention in December and April was enhanced by lower velocities and, notably, the accumulation of anthropogenic solid waste. Although waste can locally trap leaves, it represents ecosystem degradation rather than resilience, creating stagnant zones that alter oxygen dynamics and microbial processing.

This reduced or “artificial” leaf retention has significant public health implications. Rapid downstream transport of organic matter increases nutrient loading, promoting eutrophication and the proliferation of fecal indicator bacteria [39]. Simultaneously, leaves trapped by anthropogenic debris create stagnant pools that facilitate the breeding of disease vectors such as *Aedes aegypti* [45]. Hydrological flashiness and impervious urban surfaces further increase flood exposure, potentially mobilizing contaminated sediments and enhancing human contact with pathogens. Ultimately, low retention serves as a functional indicator of a “short-circuited” ecosystem with reduced capacity for nutrient spiraling [46]. Strategies must move beyond simple pollution control to incorporate the restoration of riparian structural integrity, which is essential for sustaining detritus-based food webs and maintaining the overall functionality of altered “wetscapes” [47]. Temporal patterns in leaf retention reflect the interplay of hydrology, physical obstacles, and ecological factors [48]. Lower flow velocities and obstacle accumulation in December, February, and April facilitated higher retention [49], although reduced discharge may also limit resource availability for detritivores and decomposers [50,51,52]. Retention is also influenced by stream velocity, substrate heterogeneity, and woody debris, which enhance retention efficiency across both temperate and tropical streams [53,54,55,56,57].

These patterns highlight the combined effect of streambed heterogeneity and hydrological variation on litter dynamics. Seasonal shifts in temperature, flow, and precipitation influence organic matter breakdown and retention in tropical streams [53,54]. Despite conducting experiments during the rainy season to reduce precipitation variability, discharge still fluctuated significantly, confirming that hydrological conditions dominate organic matter retention. In tropical freshwater systems, the relationship between climate and organic matter dynamics often differs from temperate regions. While temperature is a dominant driver in temperate streams, precipitation is typically the major control across tropical biomes, explaining most litter inputs and storage in the Atlantic Forest, Amazon, and Cerrado savanna [53]. However, in urbanized tropical streams like the Bindá, this natural relationship is disrupted.

Channelization is a known factor that accelerates water transport [55], and this was evident in the Bindá stream, where the artificial channel structure likely enhanced downstream leaf drift and reduced retention [56]. Urbanization affects not only the aesthetic but also the ecological functionality of aquatic systems [58]. The observed low retention (≤33%) reflects the lack of environmental complexity due to intense anthropogenic alteration, confirming previous assessments of high anthropogenic land use in the Bindá watershed [35]. Forested Amazonian streams retain leaves up to 10 times more effectively than deforested ones [30,59,60], with reported retention exceeding 84% in >70% of cases [61].

Trash dams partially compensate for lost natural obstacles but do not replicate ecosystem functions of woody debris, creating anoxic zones and impairing ecological processes, which underscores the need to remove waste and restore riparian complexity [20].

Retention reductions similar to Bindá Stream have been observed in urban streams in Malaysia, Indonesia, and Nigeria, where impervious surfaces and riparian loss reduced organic matter retention by 40–70% [62]. This supports that the Bindá Stream findings are generalizable to other tropical urban catchments with comparable land-use and hydrological conditions.

Riparian buffers are crucial: they preserve leaf retention, maintain ecological processes, and regulate water quality [51,63,64,65,66]. Larger woody debris stability, aquatic plant dispersal, and three-dimensional hydrological connectivity also govern material transport in Amazonian streams [67,68,69]. In the Bindá, connectivity is compromised by channelization and the loss of riparian integrity, leading to a fragmented and dysfunctional ecosystem [18].

From a public health side, degraded urban headwaters undermine ecosystem services that naturally regulate water quality, flooding, and pollutant exposure [70,71]. Riparian restoration and hydrological connectivity function as preventive environmental health interventions [1]. Evidence shows that enforcing riparian buffers, green infrastructure, and community-based watershed management reduces nutrient export, suspended solids, fecal indicators, and vector breeding [1,20,72,73].

Our findings provide actionable evidence for municipal urban planning in Manaus, particularly regarding riparian buffer enforcement, solid waste management, and community-based watershed stewardship. Empirical evidence from urban watersheds in southern Brazil demonstrates that restoring 15–30 m riparian buffers significantly reduces nutrient export, suspended solids, and fecal indicator bacteria [20]. Similarly, green infrastructure projects in Medellín, Colombia, resulted in measurable improvements in water quality and reductions in vector breeding habitats [1].

For Manaus, enforcing the Brazilian Forest Code [74], which mandates permanent preservation areas (PPAs) along watercourses, could reduce organic matter export and mitigate flash flooding [73]. Restoration of native riparian vegetation would increase leaf retention capacity, stabilize streambanks, and reduce downstream pollutant transport [1].

Therefore, the accumulation of anthropogenic debris in Bindá Stream reflects upstream waste management failures. Strengthening municipal and neighborhood-level waste collection, along with debris interception systems, can effectively reduce stream obstruction and stagnant pools, mitigating ecological and public health risks [1].

Participatory watershed programs in Southeast and Northeast of Brazil demonstrated that community-based monitoring reduced illegal dumping and improved riparian vegetation maintenance [75]. Community stewardship programs also increase compliance with environmental regulations. In Manaus, integrating environmental education with primary health outreach programs could simultaneously address vector control and watershed protection.

Riparian restoration is not solely an ecological intervention but a preventive public health strategy [1]. Evidence from urban river rehabilitation projects in India and Indonesia indicates that improved water flow and reduced organic stagnation correlate with lower vector density and reduced diarrheal disease incidence [45]. Thus, integrating watershed management into municipal health planning frameworks could enhance disease prevention strategies in vulnerable peri-urban communities.

Limitations of this study include: (i) float-derived velocities may underestimate turbulence effects; (ii) painted leaves may alter settling; (iii) sediment transport and continuous microbial monitoring were not included. Future research should integrate microbial and epidemiological data to quantify direct health impacts of urban stream degradation.

### Policy Implications for Urban Stream Management in Manaus

The present findings demonstrate that hydrodynamic intensity strongly governs organic matter transport in the studied urban stream. The observed low retention capacity under high discharge conditions suggests a limited buffering potential against downstream material export, a characteristic hallmark of the “urban stream syndrome” [38]. Although this study did not directly quantify water-quality or epidemiological indicators, extensive research in tropical and subtropical systems has shown that riparian buffer restoration significantly reduces fecal coliform concentrations, decreases nutrient and sediment loads, and stabilizes streambanks against urban flash flooding [33,75].

In this context, our results provide functional evidence that maintaining or increasing structural complexity in riparian zones can enhance hydraulic roughness and improve retention capacity, particularly under moderate flow conditions [35]. For municipal authorities in Manaus, the following interventions are supported by both our empirical findings and the established ecohydrological literature:

1. Riparian Buffer Zoning: Establishing and strictly enforcing minimum vegetated buffer widths along urban streams is essential to increase hydraulic resistance and filter direct runoff [33]. Such zoning acts as a critical interface that mitigates the “flashy” hydrological response typical of the region’s intense rainfall [43].
2. Restoration of Structurally Diverse Riparian Strips: Management should prioritize mixed-species planting—incorporating both canopy and understory layers—to enhance vertical complexity. This diversity increases the “structural template” necessary for organic matter entrapment and flow deceleration [34,36].
3. Stormwater Management Integration: To improve retention efficiency, it is vital to reduce the connectivity of impervious surfaces to the stream network through green infrastructure, such as bioswales and infiltration systems [40]. These nature-based solutions help lower peak discharge, allowing natural retention structures to function effectively.
4. Solid Waste Management Enforcement: Given that anthropogenic debris partially influenced retention patterns in our study, prioritizing waste reduction is crucial to prevent “dysfunctional retention”. Such artificial dams create stagnant zones that exacerbate anoxia and degrade biogeochemical processing [7].
5. Community Engagement Programs: Urban stream stewardship initiatives are necessary to reduce litter deposition and ensure the long-term maintenance of riparian vegetation, fostering a sense of local ownership over urban “wetscapes” [47].

Rather than proposing restoration as a direct public-health intervention, we frame riparian management as a structural hydrological strategy. By restoring the physical complexity of the stream-riparian interface, managers can indirectly facilitate the recovery of ecosystem services, such as nutrient spiraling and pathogen attenuation, which are fundamental to the health of both urban watersheds and population [39,75].

## 5. Conclusions

This study shows that the urbanized Bindá Stream has a very limited capacity to retain leaf litter, with retention consistently below 33%, primarily due to channel modification, altered hydrological conditions, and the low structural complexity of riparian vegetation. The presence and increasing influence of exotic and cultivated species and the scarcity of natural retention structures, such as large woody debris, strongly constrained organic matter dynamics in this urban headwater stream.

Seasonal variations in discharge and flow velocity were key drivers of leaf transport, with higher flows promoting downstream export and lower flows favoring localized retention. However, even under reduced discharge, retention remained low, highlighting that hydrological conditions alone cannot compensate for the loss of riparian structural complexity.

Anthropogenic alterations, including channelization and solid waste accumulation, modified retention patterns without restoring ecological function. Although debris may locally increase retention, this process reflects ecosystem degradation rather than resilience and is associated with declining water quality.

Beyond ecological impacts, reduced organic matter retention compromises natural processes that regulate water quality and hydrological stability, increasing the downstream transport of pollutants and the risk of human exposure during flood events. In rapidly urbanizing tropical cities, such degradation undermines ecosystem services that are critical for environmental and public health.

Importantly, our results explicitly support the three hypotheses initially proposed: (1) reduced riparian complexity and increased flow variability limit organic matter retention; (2) anthropogenic debris partially compensates for natural retention but creates ecologically dysfunctional retention structures; and (3) diminished retention reduces the buffering capacity of urban streams, enhancing downstream material export under high-flow conditions. Together, these findings provide a strong functional basis for prioritizing riparian restoration and integrated watershed management in tropical urban settings.

Restoring riparian vegetation and stream structural complexity should therefore be prioritized as a nature-based solution that enhances organic matter retention, improves water quality, and reduces reliance on energy-intensive grey infrastructure. Integrating urban stream restoration into land-use planning and environmental health policies is essential to promote climate adaptation, ecosystem resilience, and healthier urban environments in the Global South.

## Figures and Tables

**Table 1:** Hydrological and physical parameters measured during the leaf transport experiment in the Bindá Stream (Manaus, Brazil).

Year	Month	Flow Velocity (m s^−1^)	Width (m)	Depth (m)	Discharge (m^3^ s^−1^)	Precipitation (mm)
2017	November	0.35 ± 0.14	3.17 ± 0.36	0.35 ± 0.06	0.39 ± 0.19	0.00 ± 0.01
	December	0.43 ± 0.22	3.10 ± 0.33	0.22 ± 0.08	0.26 ± 0.63	3.44 ± 5.50
2018	January	0.43 ± 0.18	2.88 ± 0.21	0.38 ± 0.08	0.44 ± 0.11	1.20 ± 2.10
	February	0.27 ± 0.17	2.86 ± 0.36	0.34 ± 0.07	0.24 ± 0.10	1.72 ± 3.06
	March	0.45 ± 0.18	2.87 ± 0.21	0.38 ± 0.07	0.46 ± 0.11	1.65 ± 2.63
	April	0.33 ± 0.20	3.48 ± 0.37	0.34 ± 0.06	0.35 ± 0.10	1.35 ± 2.53

Values are expressed as means ± standard deviation.

**Table 2:** Tree and shrub species recorded in the adult (arboreal) and regeneration strata of the riparian forest along the Bindá Stream, Manaus, AM, Brazil.

Family	Species	Native/Introduced	Adult Abundance	Regeneration Abundance
Fabaceae	*Clitoria fairchildiana* R.A. Howard	Native	41	0
Fabaceae	*Inga edulis* Mart.	Native	7	15
Fabaceae	*Senna alata* (L.) Roxb.	Native	2	0
Anacardiaceae	*Anacardium occidentale* L.	Native	1	0
Anacardiaceae	*Spondias mombin* L.	Native	7	0
Anacardiaceae	*Mangifera indica* L.	Introduced	0	23
Myrtaceae	*Psidium guajava* L.	Naturalized	1	1
Myrtaceae	*Syzygium cumini* (L.) Skeels	Introduced	10	26
Myrtaceae	*Syzygium malaccense* (L.) Merr. and L.M. Perry	Introduced	0	8
Rubiaceae	*Genipa americana* L.	Native	0	1
Arecaceae	*Euterpe oleracea* Mart.	Native	32	26
Arecaceae	*Mauritia flexuosa* L.f.	Native	0	1

**Table 3:** Composition of herbaceous species recorded along the riparian margins of the Bindá Stream during the dry and rainy seasons, highlighting seasonal variation in lateral vegetation cover.

Family	Species	Dry Season	Rainy Season
Commelinaceae	*Commelina erecta* L.		X
Cyperaceae	*Cyperus odoratus* L.		X
Cyperaceae	*Cyperus difformis* L.		X
Cyatheaceae	*Cyathea pungens* (Willd.) Domin		X
Euphorbiaceae	*Phyllanthus niruri* L.		X
Euphorbiaceae	*Euphorbia prostrata* Aiton		X
Fabaceae	*Mimosa pudica* L.		X
Lomariopsidaceae	*Cyclopeltis semicordata* (Sw.) J. Sm.		X
Poaceae	*Eleusine indica* (L.) Gaertn.		X
Poaceae	*Digitaria ciliaris* (Retz.) Koeler		X
Poaceae	*Brachiaria plantaginea* (Link) Hitchc.		X
Poaceae	*Pennisetum purpureum* Schumach.	X	
Polypodiaceae	*Phlebodium decumanum* (A.M. Evans) M. Kessler and A.R. Sm.		X
Pteridaceae	*Pteris ensiformis* Burm. f.		X

**Table 4:** Number and proportion of leaves retained and transported over a 30 m reach of the Bindá Stream, Manaus, Amazonas, Brazil.

Year	Month	Leaves Retained (n)	% Retained	Leaves Transported (n)	% Transported
2017	November	48	24%	152	76%
	December	65	33%	135	67%
2018	January	18	9%	182	91%
	February	57	29%	143	71%
	March	27	14%	173	86%
	April	62	31%	138	69%

## Data Availability

The data presented in this study are available on request from the corresponding author. The data are not publicly available due to institutional and project-related restrictions.

## Abbreviations

The following abbreviations are used in this manuscript:

APP	Permanent Preservation Area
ANOVA	Analysis of Variance
AT	Total cross-sectional area of the stream
CPOM	Coarse Particulate Organic Matter
DOI	Digital Object Identifier
LR	Log-rank test
m	Meter
m s^−1^	Meters per second
m^2^	Square meters
m^3^ s^−1^	Cubic meters per second
PVC	Polyvinyl Chloride
Q	Stream discharge
R	R statistical software environment
SDG	Sustainable Development Goal
SDG 3	Good Health and Well-being
SDG 7	Affordable and Clean Energy
SDG 13	Climate Action
χ^2^	Chi-square statistic
